# Photovoltaic Effect Produced in Silicon Solar Cells by X- and Gamma Rays^1^

**DOI:** 10.6028/jres.064A.029

**Published:** 1960-08-01

**Authors:** Karl Scharf

## Abstract

The open-circuit voltage and photocurrent produced in a silicon solar cell by X- and gamma rays were measured as a function of exposure dose rate, cell temperature, angle of incidence of radiation, and photon energy. This photoresponse was stable and proportional to the exposure dose rate, which was applied up to a maximum of 1.8×10^6^ roentgen per minute for X-rays and 4×10^2^ roentgen per minute for Co^60^ gamma rays. At an exposure dose rate of 1 roentgen per minute the response was of the order of 10^−5^ volt for the open-circuit voltage and 10^−8^ ampere for the photocurrent. At high exposure dose rates of Co^60^ gamma rays, radiation damage became apparent. The temperature dependence of the photoresponse was controlled by the temperature dependence of the cell resistance. The directional dependence of the photoresponse varied with the quality of radiation and for Co^60^ gamma rays was very small for angles from 0° to 70°. The photoresponse decreased with increasing photon energy but changed only little between 200 and 1,250 kilo electron volts. The ratio of the response to X-rays of 38 kilo electron volts effective energy and that to Co^60^ gamma rays was approximately 6:1. An approximate value of the thickness of the effective *p–n* junction layer is deduced from the energy dependence.

## 1. Introduction

The investigation reported in this paper was carried out in order to test the suitability of silicon solar photocells for dosimetry of X- and gamma rays.

Silicon solar cells are photovoltaic cells of the *p–n* junction type. When irradiated with photons of an energy sufficient to produce electron-hole pairs in silicon, a photocurrent is produced without application of an external electrical power supply. The photocurrent or the photovoltage developed between the two sides of the junction can be used as a measure of the intensity of the incident radiation.

Photovoltaic cells are frequently used for measurements of the intensity of visible light. Only few quantitative investigations have been made of the response of photovoltaic cells to X- and gamma rays. More detailed investigations were carried out on cuprous-oxide [1, 2]^2^ and selenium [1, 3, 4] cells of the metal-semiconductor contact type and on germanium [5] and gallium arsenide [6] *p–n* junction photocells. Large area selenium cells had the greatest overall sensitivity but showed a very slow photoresponse to X-rays, similar to that observed in photoconductive cells. Photovoltaic *p–n* junction cells were less sensitive than selenium cells but had response times of the order of milliseconds [6].

No detailed investigation of the response of silicon solar cells to X- and gamma rays has been reported. The investigation of these cells seemed to be promising for different reasons. The silicon solar cell is a large area photovoltaic cell which has a high current output and a short response time [7]. It was also expected that due to the low atomic number of silicon (Z_Si_=14), the response of silicon solar cells would be less dependent on incident photon energy than that of other photovoltaic cells.

Measurements are reported of the response of silicon solar cells to X- and gamma-rays as a function of (1) the exposure dose rate of the radiation, (2) the cell temperature, (3) the angle of incidence of the radiation, and (4) the incident photon energy.

## 2. General Considerations

**Figure 1:**
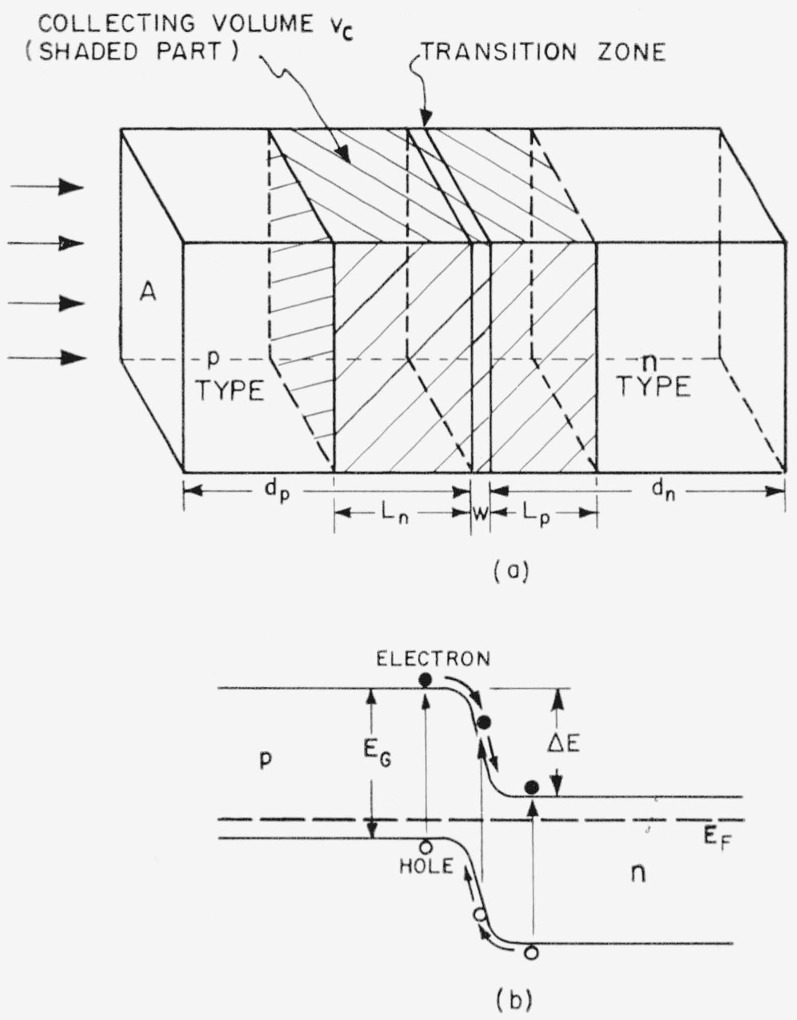
*(a)* Geometry of *p–n* junction photocell showing the photoelectric effective collecting volume *v_c_* determined by the minority carrier diffusion lengths *L_n_* and *L_p_*. *(b)* Equilibrium configuration of electron energy bands in a *p–n* junction.

A silicon solar cell consists of a large area *n*-type silicon disk with a very thin *p*-type layer on its surface [8]. In balancing an equilibrium between the different carrier concentrations in the *p*-type and *n*-type silicon, a strong electrostatic field is established in the transition zone (fig. 1). The electric junction field is due to a potential difference with the higher potential on the side of the *n*-type layer. This potential difference, sometimes called the “diffusion potential,” is of the order of 1 v in silicon. This can be seen from the configuration of the electron energy bands in the *p–n* junction as shown in figure 1. In the equilibrium state, the Fermi level *E_F_* is constant throughout the *p*- and *n*-type layers and the barrier height Δ*E* is slightly smaller than the energy gap *E_G_*, which is 1.1 ev for silicon.

The *p–n* junction has a current-voltage characteristic of an electric rectifier with the forward current flowing from the *p*-type to the *n*-type layer. If the *p–n* junction is irradiated with photons of an energy larger than *E_G_*, electron-hole pairs are produced which are separated by the junction field. Electrons are driven to the *n*-type and holes to the *p*-type layer. In this way a voltage difference is produced between the two sides of the junction, the *p*-side becoming positive and the *n*-side negative. This photovoltage biases the *p–n* junction in the forward direction and opposes the diffusion voltage.

The current carriers produced by the radiation and separated by the junction field represent the generated photocurrent in the reverse direction of the junction. In restoring the equilibrium state, the photovoltage produces a current through the junction in the forward direction. Provided that no external load is connected to the cell, the open- circuit voltage (photoelectromotive force) reaches a steady value at which the generated photocurrent equals the junction current produced by the photovoltage. If a load resistance is connected across the junction, part of this current flows through the external load and the photovoltage is reduced.

**Figure 2:**
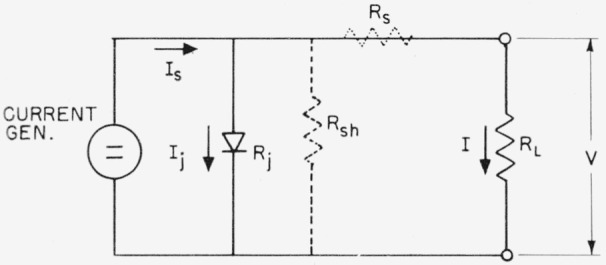
Equivalent circuit of *p–n* junction photocell.

The electrical characteristic of a *p–n* junction photovoltaic cell can be derived by considering the equivalent circuit shown in figure 2. The photocell is represented as a constant current generator.

The generated photocurrent *I_s_* divides into the junction current *I_j_* and the external load current *I. R_j_* is the voltage dependent junction resistance in the forward direction and *R_L_* is the load resistance. It may be assumed that the series cell resistance *R_s_* and shunt resistance *R_sh_* need not be considered if *R_s_<<R_L_<<R_sh_*, a condition usually fulfilled.

According to the theory of the *p–n* junction [9], one obtains

$$I_{j}=I_{0}[\exp(qV/kT)-1].$$ (1)

*I*_0_ is the saturation current of the junction in the reverse direction, *q* the electronic charge, *k* the Boltzman constant, *T* the absolute temperature, and *V* the photovoltage. The photocurrent *I* measured in the external load is then

$$I=I_{s}-I_{j}=I_{s}-I_{0}[\exp(qV/kT)-1].$$ (2)

From this equation one obtains for the photovoltage measured across the cell terminals

$$V=\frac{KT}{q}\ln\left(\frac{I_{s}-I}{I}+1\right).$$ (3)

Two special cases are usually considered:

- Open circuit condition:
  $$\begin{array}{c} R_{L}=\infty ;\;I=0;\;I_{s}=I_{j}\\ V\equiv V_{0}=\frac{KT}{q}\ln\left(\frac{I_{s}}{I_{0}}+1\right). \end{array}$$ (4)
  For (*I_s_/I*_0_)<< 1, one obtains
  $$V_{0}=\frac{KT}{q}\cdot \frac{I_{s}}{I_{0}}$$ (4a)
- Short circuit condition:
  $$R_{L}=0;\;I_{j}=0;\;V=0;\;I=I_{s}$$ (5)

The open-circuit voltage *V*_0_ and the generated current *I_s_*, usually called the short-circuit current, are considered as characteristic measures for the photoresponse of the cell.

The theory of the photovoltaic effect assumes that, in addition to carriers produced inside the junction, all minority carriers produced outside it but able to reach the junction by diffusion, are contributing to the photocurrent. The photoelectric effective area is therefore determined by the diffusion lengths of the minority carriers, the diffusion length being defined as the average distance that carriers diffuse before recombining.

If the mean free path of the radiation (equal to the reciprocal of the absorption coefficient measured in cm^−1^) is large compared with the diffusion lengths one obtains for the width *L* of the effective area

$$L=L_{n}+L_{p}+W.$$ (6)

*L_n_* is the diffusion length of electrons in the *p*-type layer, *L_p_* the diffusion length of holes in the *n*-type layer, and *W* is the width of the junction (fig. 1). Usually *W* is very small compared with *L_p_* and *L_n_* and can be neglected. In solar cells *d_p_<L_n_*, if *d_p_* is the thickness of the *p*-layer. The value of *L* is therefore approximately

$$L=L_{p}+d_{p}.$$ (6a)

The generated current *I_s_* will comprise all minority carriers produced by radiation in the collecting volume

$$v_{c}=A(L_{p}+d_{p})=AL,$$ (7)

provided that the whole junction area *A* is being irradiated. It has also been shown that the saturation current *I*_0_ will comprise all minority carriers thermally generated in the same collecting volume *v_c_.*

Considering eqs (2) and (3) one obtains [10]

$$I=qAg_{0}L-qAgL\prime [\exp(qV/kT)-1]$$ (8)

and

$$V=\frac{kT}{q}\;\ln\;\left(\frac{qAg_{0}L-I}{qAgL\prime }+1\right)$$ (9)

if

$$gL\prime =g_{p}L_{p}+g_{n}d_{p}$$ (10)

where *g*_0_ is the rate of generation of electron-hole pairs due to radiation and is equal to their number produced by the radiation per cubic centimeter per second, and *g_p_* and *g_n_* are the thermal generation rates of holes in the *n*-layer and electrons in the *p*-layer, respectively.

From eqs (8) and (9) one obtains for the generated or short circuit current

$$I_{s}=qAg_{0}L=qg_{0}v_{c}$$ (11)

and for the open-circuit voltage

$$V_{0}=\frac{kT}{q}\;\ln\;\left(\frac{g_{0}L}{gL\prime }+1\right)$$ (12)

For (*g*_0_*L/gL*′)<<1, the approximation can be made

$$V_{0}=\frac{KT}{q}\cdot \frac{g_{0}L}{gL\prime }.$$ (12a)

The above relations were derived for the photovoltaic effect produced by visible light but are also valid for irradiation with X- and gamma rays. In the case of visible light each absorbed photon excites one electron from the valence band into the conduction band and thus produces one electron-hole pair. High-energy photons will mostly ionize low lying atomic energy levels (*K*- and *L*-levels) producing photoelectrons or will interact with atomic electrons mainly in higher levels by a Compton scattering process producing recoil electrons and Compton photons. Pair production will have to be considered for photons of energy larger than 1.02 Mev. High-energy electrons, produced in these interactions, transfer part of their energy to the crystal lattice of silicon, but lose most of their energy by impact ionization thus producing low energy current carriers.

High-energy electrons, which pass the *p−n* junction are negligibly influenced by the low potential barrier of approximately 1 ev. Only low-energy carriers are acted upon by the junction field in such a way that they contribute to the photocurrent. Equations (8) and (9) are, therefore, also applicable in the case of high-energy radiation, provided that *g*_0_ refers to the generation rate of low-energy carriers produced by the high-energy electrons. The collecting volume *v_c_* will be the same as in the case of visible light.

## 3. Experimental Procedure

The photocells investigated were commercial round silicon solar cells^3^ with a sensitive area of 7.9 cm^2^. The silicon disk of the cell was approximately 0.7 mm thick and was encapsulated in a metal casing. The front of the cell was closed with a glass window 1.25 mm thick. The whole cell was wrapped in black insulation tape in order to prevent the access of light.

The X-ray sources were a 250-kv Machlett tube with an inherent filtration of 3-mm Al and a 50-kv Machlett beryllium window-type tube with an inherent filtration of 1-mm Be. The tubes were tungsten target tubes operated by a stabilized constant voltage supply. A calibrated 100-curie Cs^137^ and a calibrated 200-curie Co^60^ source were used as gamma ray sources.

Unless otherwise stated, exposure dose rates were measured with Victoreen *r*-meters which had been calibrated for each investigated type of radiation against the NBS standard free air chambers.^4^ All measurements were carried out with an irradiation of the whole sensitive cell area.

The open circuit voltage was measured by an electric compensation method. The photocurrent was determined either directly with a microammeter or sensitive galvanometer or by measuring the voltage drop across a known load resistance with a potentiometer.

## 4. Results and Discussion

### 4.1 Exposure Dose Rate Dependence

**Figure 3:**
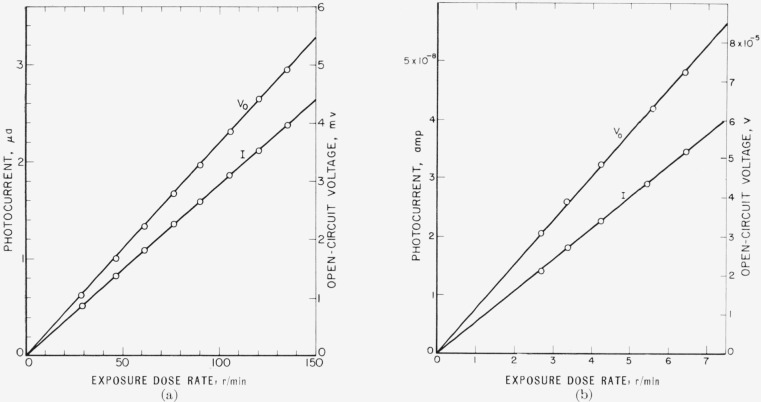
Dependence of open-circuit voltage *V_o_* and photocurrent *I* on exposure dose rate measured at room temperature *(a)* with 250-kv unfiltered X-rays (load resistance *R_L_*=890 ohms), and *(b)* with *Co^60^* gamma rays (*R_L_*=532 ohms).

Figure 3 shows the open-circuit voltage *V*_0_ and the photocurrent *I* as a function of the exposure dose rate for 250 kv unfiltered X-rays (fig. 3a) and Co^60^ gamma rays (fig. 3b). The exposure dose rate of the X-rays was measured with the NBS 250 kv standard free air chamber and was varied by changing the tube current. In the case of Co^60^ gamma rays, the exposure dose rate was varied by changing the distance between source and photocell. The exposure dose rates at different distances were obtained from the calibration curve of the source. Exposure dose rates were increased up to approximately 10^4^ r/hr for the X-rays and up to 400 r/hr for the Co^60^ gamma rays.

Open circuit voltage and photocurrent produced by X- and gamma rays were proportional to the exposure dose rate. The photoresponse remained constant during the irradiation and was reproducible within the accuracy of the measuring instruments and the constancy of the radiation source.

**Figure 4:**
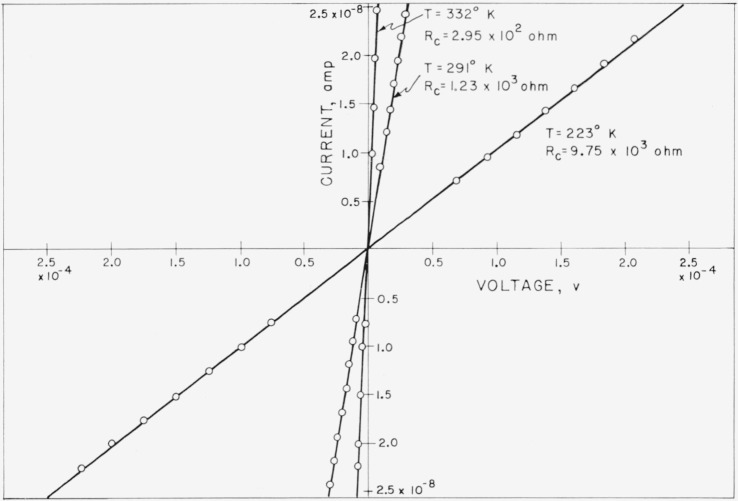
Current-voltage characteristics of the investigated silicon solar cell measured at different cell temperatures *T*, with indication of respective cell resistance *R_c_*.

In order to obtain the generated or short circuit current *I_s_* from the measured photocurrent *I*, the current-voltage characteristic of the cell must be known. Figure 4 shows the current-voltage characteristics of the photocell measured at different temperatures for very low voltages. The characteristic is linear and its slope gives the zero voltage resistance *R_c_* of the photocell. At the low photovoltages encountered in this investigation, the cell resistance may be assumed as voltage independent and equal to the zero voltage junction resistance *R_j_*, provided that *R_s_* and *R_sh_* need not be considered. Substituting in eq (2) the relations

$$I_{j}=V/R_{j}\;\text{and}\;I=V/R_{L}$$

one obtains

$$I_{s}=I(1+R_{L}/R_{j})$$ (13)

and

$$V_{0}=I_{s}R_{j}$$ (14)

when *R_L_* is the load resistance. Equation (13) shows that *I_s_* differs from *I* by a constant factor, which is determined by the ratio *R_L_/R_j_.* According to eq (14)*V*_0_ is proportional to *I_s_* and both should show the same functional behavior, if R*_j_* is constant.

The junction resistance *R_j_* of the investigated cell was approximately 1,000 ohms at room temperature. The values of the photocurrent *I* shown in figure 3 have, according to eq (13), to be multiplied by a factor of 1.89 (*R_L_*=890 ohms) for X-rays (fig. 3a and 1.53 (*R_L_*=532 ohms) for gamma rays (fig. 3b) in order to obtain the short circuit currents *I_s_.* This means that measurements shown in figure 3 indicate the proportionality between the short circuit current *I_s_* and exposure dose rate as well. In case the series resistance *R_s_* and shunt resistance *R_sh_* must be taken into account, the impedance of the combined resistances needs to be considered. The proportionality between *I* and *I_s_* and *V*_0_ and *I_s_* would still be maintained.

**Figure 5:**
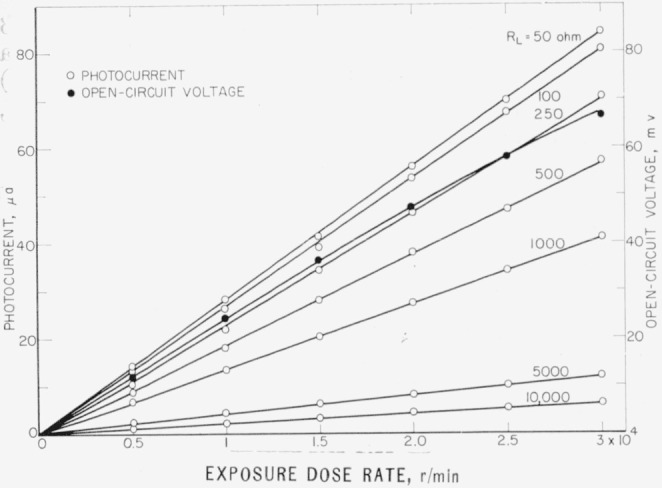
Exposure dose rate dependence of open-circuit voltage and photocurrent measured at room temperature with different load resistances at high exposure dose rates of 50-kv unfiltered X-rays obtained from a beryllium-window-type tube.

Measurements were further carried out with X-rays obtained from a beryllium window-type tube operated at 50 kv (fig. 5). Because of the small inherent filtration of this tube, the radiation comprised a greater amount of low-energy radiation which was strongly absorbed in the cell. *V*_0_ and *I* were measured at high exposure dose rates up to 1.8×10^6^ r/hr. The attenuation of the radiation in the glass window of the cell was taken into account. Photocurrents increased linearly with exposure dose rate with different load resistances up to 10^4^ ohm. The open circuit voltage showed for higher exposure dose rates a nonlinear dependence following the logarithmic relation of eq (4). The highest response obtained in these measurements with R*_L_*=50 ohm was approximately *I*=90 *μ*a and *V*_0_=70 mv. At 1.8×10^6^ r/hr exposure dose rate, the maximum matched load power output was 1.7 *μ*w. This corresponds to a conversion efficiency of approximately 0.02 percent. For higher energy radiation the conversion efficiency decreases due to the reduced absorption of the radiation.

The maximum photocurrent observed at these high exposure dose rates remainted constant during irradiation over several hours. The open-circuit voltage showed a slow decrease in time of irradiation, which has apparently due to an increase of the cell temperature. When the radiation was shut off and the cell allowed to cool down, the open-circuit voltage recoved to the original value at a subsequent irradiation.

Open-circuit voltage and photocurrent produced by gamma rays obtained from a water-shielded 2,000 curie Co^60^ source at an exposure dose rate of approximately 6×10^5^ r/hr, showed a considerable decay with time of irradiation. Simultaneously with the decay of the photoresponse the electrical resistance of the cell decreased. Such transient response, apparently due to radiation damage, was not observed in measurements with gamma rays at low exposure dose rates and is also not in agreement with measurements reported by other authors. Moody et al. [11] found a constant response of silicon solar cells to Co^60^ gamma rays at an exposure dose rate of approximately 10^5^ r/hr. Loferski and Rappaport [12] found that the photoresponse of silicon solar cells to visible light remained unaffected by simultaneous irradiation with 750 kv and 2 Mev X-rays. The response of silicon solar cells to high-energy radiation at high exposure dose rates is being further investigated by the author.

### 4.2. Temperature Dependence

*V*_0_ and *I* produced by Cs^137^ gamma rays were measured for different cell temperatures between −50° C and 60° C. The silicon solar cell was placed inside a wooden box in which the temperature could be regulated by means of a thermostat. Dry ice was used for obtaining temperatures below room temperature. The gamma-ray source was placed outside the box.

**Figure 6:**
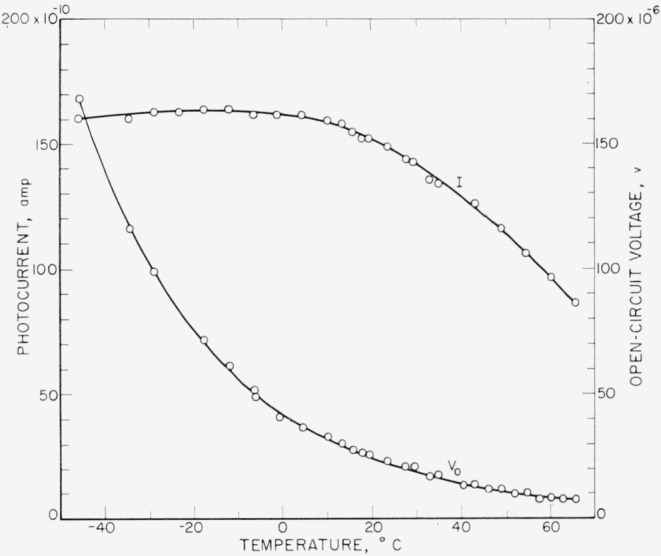
Temperature dependence of open-circuit voltage *V_o_* and photocurrent *I* (*R_L_*=532 ohms) produced by Cs^137^ gamma rays.

The results of these measurements are shown in figure 6. V_0_ decreased nearly exponentially with increasing temperature. The photocurrent *I* measured with a load resistance of *R_L_*=532 ohms was approximately constant up to 10° C and decreased then with increasing temperature. The temperature dependence of *V*_0_ and *I* will, according to eqs (13) and (14), be determined by the temperature dependence of *R_j_.* The generated current *I_s_* may be assumed to be independent of temperature, because the generation rate *g*_0_ and the diffusion length *L_P_* (eq (11)) remain approximately constant over a limited range of temperature. *R_j_* decreases nearly exponentially with increasing temperature causing the exponential decrease of *V*_0_ (eq (14)). The temperature dependence of *I* is determined by the ratio *R_L_/R_j_* (eq (13)). *R_L_/R_j_* increases with increasing temperature and the photocurrent therefore decreases. With decreasing temperature the ratio *R_L_/R_j_* decreases and the photo current increases approaching the value of *I_s_.* However, at lower temperatures the series resistance *R_s_*, originally assumed as negligibly small, increases too and eq (13) has to be modified as,

$$I_{s}=I\;\left(1+\frac{R_{L}+R_{s}}{R_{j}}\right).$$ (13a)

The temperature dependence of *I* is now determined by the ratio (*R_L_+R_s_*)*/R_j_.* The rate of increase of *I* with decreasing temperature is reduced and I reaches a maximum at low temperatures as seen in figure 6.

**Figure 7:**
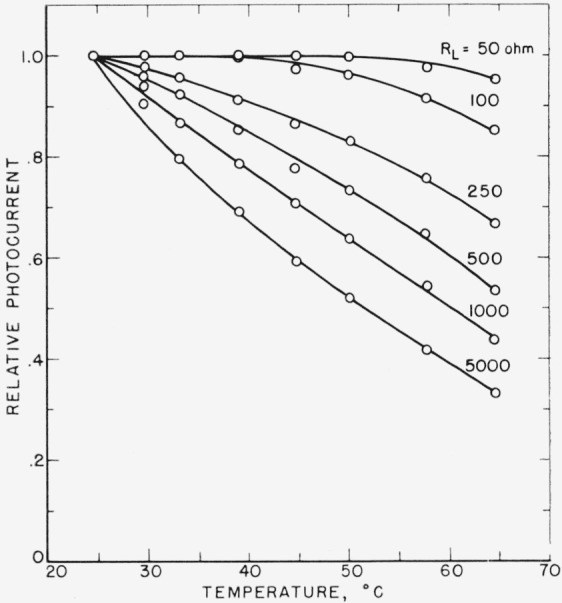
Relative change of photocurrent with cell temperature measured with 250-kv unfiltered X-rays for different load resistances.

The temperature dependence of *I* with different load resistances *R_L_* was measured with 250 kv unfiltered X-rays. The relative values of I are shown in figure 7, the photocurrent at 25° C being normalized to unity. With *R_L_*=50 ohms, the photocurrent remained constant from 25° C up to approximately 50° C and then decreased slowly at higher temperatures. With increasing *R_L_*, the temperature dependence becomes stronger. For very large *R_L_*, the photovoltage *V=IR_L_* approaches the value of *V*_0_ and the photocurrent shows a temperature dependence similar to that of *V*_0_. The influence of the load resistance on the temperature dependence of the photocurrent can in the same way, as shown above, be explained by the temperature dependence of *R_j_.*

### 4.3. Directional Dependence

**Figure 8:**
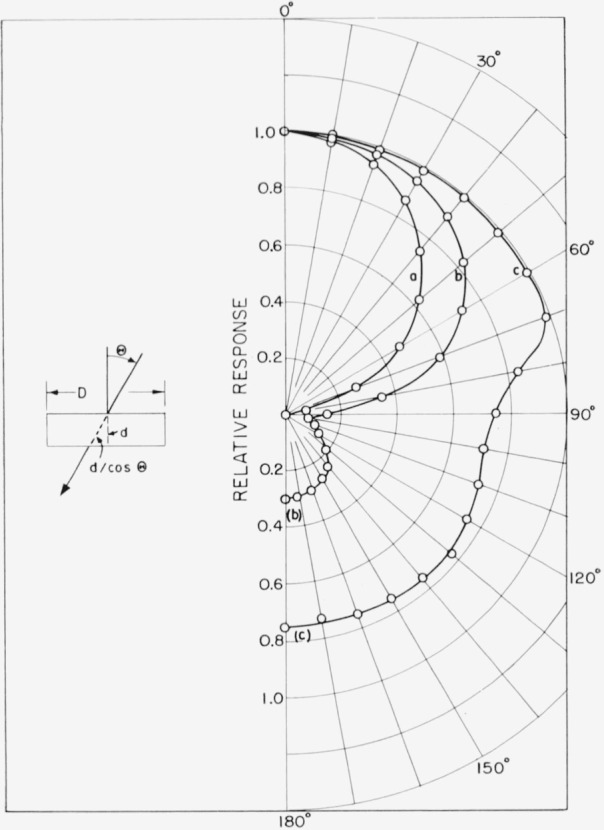
Relative values of photorespouse (open-circuit voltage *V_o_*, photocurrent *I* and generated photocurrent *I_s_*) for different angles of incidence of *(a)* 50-kv unfiltered X-rays from a beryllium-window-type tube, *(b)* 250-kv unfiltered X-rays, and *(c) Co*^60^ gamma rays.

The directional dependence of *V*_0_ and *I* measured for different qualities of radiation is shown in figure 8. The photocell was turned at different angles *θ* around an axis going through the center of the cell perpendicular to the direction of the incident radiation. *V*_0_ and *I* changed in the same way with changing *θ.* The relative values shown in figure 8 therefore apply to both.

The directional dependence measured with 50-kv unfiltered X-rays from a beryllium-window-type tube approximately follows a cosine law. There is less directional dependence for 250-kv unfiltered X-rays and the response to Co^60^ gamma rays is practically independent of *θ* up to 70°. At larger angles the attenuation in the metal casing becomes apparent. For more penetrating radiation, a response is also observed with irradiation of the backside of the cell. The directional dependence for backside irradiation is similar to that for frontside irradiation.

The directional dependence of the photoresponse can be explained by considering the energy absorbed in the effective *p–n* junction layer. The incident energy flux density will be proportional to cos *θ*, while the path length of the radiation inside the effective layer will be *d/*cos *θ*, if *d* is the thickness of the effective layer. The energy *E_a_* absorbed in this layer will therefore show a directional dependence,

$$E_{a}\propto \cos\theta [1-\exp(-\mu d/\cos\;\theta )],$$

where *μ* is the absorption coefficient of silicon. For large values of *μd*/cos *θ*, the directional dependence of *E_a_* approaches a cosine law. For *μd/*cos *θ*<<1 one can make the approximation

[1−exp(−μd/cosθ)]~μd/cosθ

and *E_a_* becomes independent of *θ.* For large values of *θ* one has to consider that the silicon disk has a finite diameter *D* and the radiation will enter the disk from the side. For angles approaching 90°, the photoresponse is a function of cos(90*−θ*) and of the disk diameter *D.*

The directional dependence of the response to Co^60^ gamma rays is in good agreement with the above relations. However they do not fully account for the directional dependence observed with low-energy X-rays. This can be explained by the fact that the radiation has to pass absorbing materials before entering the silicon disk. The energy absorbed in the effective layer is thus reduced by a direction dependent factor exp (*−μ*_1_*d*_1_/cos *θ*) if *μ*_1_ and *d*_1_ are the absorption coefficient and thickness of the absorbing materials, i.e., the glass window and metal casing of the cell. For large values of *μ*_1_*d*_1_, as in the case of low-energy X-rays, the directional dependence of the photoresponse is therefore strongly influenced by the absorption in the cell casing.

### 4.4. Energy Dependence

The photoresponse of the silicon solar cell to radiations of different photon energies was measured with heavily filtered X-rays and Cs^137^ and Co^60^ gamma rays. Tube voltages, filtrations, half value layers (HVL) in copper, and corresponding effective energies for the different investigated radiations are shown in table 1. The applied exposure dose rates were between 0.5 and 5 r/min.

**Figure 9:**
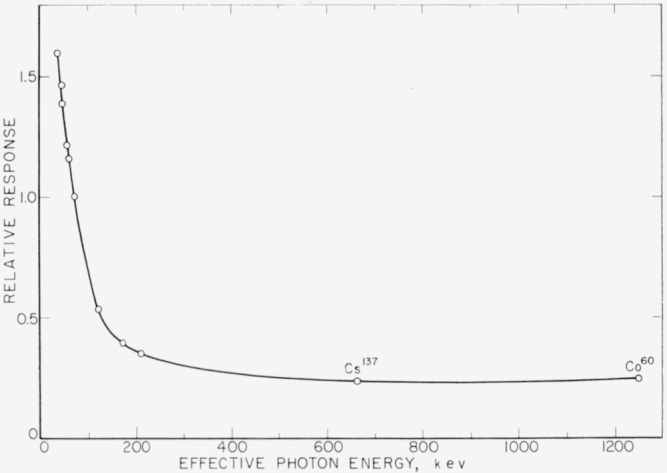
Energy dependence of relative values of photoresponse (open-circuit voltage *V_o_*, photocurrent *I*, and generated photocurrent *I_s_*) obtained from measurements at room temperature with constant load resistance. Values for 100-kv filtered X-rays (effective photon energy *hv*_eff_=70 kev) are normalized to unity.

The open-circuit voltage and photocurrent per unit exposure dose rate showed the same energy dependence. Their relative values, normalized to unity for 100-kv X-rays, are the same for both. They are shown in figure 9 as a function of the effective photon energy. The absorption of the radiation in the glass window, measured after dismantling the cell, was taken into account. The photocurrent was always measured with the same load resistance (532 ohms). The relative values given in figure 9 show, in accordance with eq (13), the energy dependence of the generated photocurrent *I_s_* as well. The normalized values measured for the 100 kv X-rays *(hv*_eff_=70 kev) per unit exposure dose rate of 1 r/min and *R_L_*=532 ohm were:

$$\begin{array}{c} {\left(V_{0}\right)}_{100}=4.12\times 10^{-5}v;{\left(I\right)}_{100}=2.34\times 10^{-8}\mathrm{amp};\\ \;{\left(I_{s}\right)}_{100}=3.58\times 10^{-8}\mathrm{amp}. \end{array}$$

The response of the silicon solar cell changes slightly between 1.25 Mev and 0.20 Mev and then rises more steeply with decreasing effective energies. The response to Co^60^ gamma rays is about 7 percent higher than to Cs^137^ gamma rays. The higher value for Co^60^ gamma rays could not be explained satisfactorily.

The energy dependence of the photoresponse is determined by the energy dependence of the generation rate *g*_0_, as seen from eqs (11) and (12). In the case of light, the number of electron-hole pairs produced in the collecting volume *v_c_* is equal to the number of photons absorbed within *v_c_.* With high-energy radiation, the photons absorbed within *v_c_* produce high-energy electrons, part of which are able to leave the collecting volume before dissipating all their energy within *v_c_.* At the same time electrons produced outside of *v_c_* enter *v_c_* and produce secondary electrons there. The net generation rate *g*_0_ is proportional to the net amount of energy dissipated in *v_c_.* In the case of electronic equilibrium, the energy lost by electrons leaving *v_c_* is compensated by the gain of energy from electrons entering *v_c_.* In this case one obtains

$$g_{0}=\frac{E_{a}}{\epsilon v_{c}}$$ (15)

where *E_a_* is the radiation energy absorbed per second in the collecting volume *v_c_*, and *ϵ* is the average energy required to produce one electron-hole pair. Considering the small dimensions of *v_c_*, the value of *g*_0_ may be assumed as constant within *v_c_.*

For the discussion of the energy dependence of the photoresponse, the relation between the absorbed energy and the generated photocurrent *I_s_* will be considered further because of the simple relation (eq 11) between *g*_0_ and *I_s_.* The radiation energy *E_a_* which is absorbed per second in *v_c_* and is transformed into electron energy is

$$E_{a}=J_{0}\;{\left(\mu_{\mathrm{en}}/\rho \right)}_{\mathrm{Si}}v_{c}\rho$$ (16)

where *J*_0_ is the intensity of the incident radiation, in erg per square centimeter and per second, *ρ* the density of silicon (2.42 g/cm^3^) and (*μ*_en_/*ρ*)_Si_ the mass energy absorption coefficient of silicon in square centimeters per gram defined as:^5^

$${\left(\mu_{\mathrm{en}}/\rho \right)}_{\mathrm{Si}}={\left(\tau /\rho \right)}_{\mathrm{Si}}+{\left(\sigma_{a}/\rho \right)}_{\mathrm{Si}}+{\left(\kappa_{a}/\rho \right)}_{\mathrm{Si}};$$ (17)

(*τ*/*ρ*)_Si_ *(σ*_a_/*ρ*)_Si_ and (*κ*_a_/*ρ*)_Si_, are the respective mass absorption coefficients referring to photoelectric absorption, energy absorbed by Compton scattered recoil electrons and absorption by pair production. In the investigated energy range, (*κ*_a_/*ρ*)_Si_ is zero except for Co^60^ gamma rays.

According to the definition of the roentgen unit, the following relation [13] can be substituted in eq (16):

$$J_{0}=\frac{87.7R}{{\left(\mu_{\mathrm{en}}/\rho \right)}_{\mathrm{air}}}\;\mathrm{erg}/\sec,{\mathrm{cm}}^{2}$$ (18)

if *R* is the exposure dose rate in roentgens per second and (*μ*_en_/*ρ*)_air_ the mass energy absorption coefficient of air. Substituting the value of *g*_0_ from eq (15) in eq (11) and considering eqs (16) and (18) one obtains the generated photocurrent *I_s_* by expressing the absorbed energy in electron volts and the electronic charge in coulombs as

$$I_{s}=2.11\times 10^{-5}R\left(v_{c}/\epsilon \right)\;f\;\mathrm{amp}.$$ (19)

where *f* is the ratio (*μ*_en_/*ρ*)_Si_/(*μ*_en_/*ρ*)_air_. For non-monochromatic filtered X-rays the energy spectrum of the radiation has to be considered in calculating *f* as

$$f=\frac{\int_{0}^{E_{\max}}J_{0}\left(E\right)\;{\left[\mu_{\mathrm{en}}\left(E\right)/\rho \right]}_{\mathrm{Si}}dE}{\int_{0}^{E_{\max}}J_{0}\left(E\right)\;{\left[\mu_{\mathrm{en}}\left(E\right)/\rho \right]}_{\mathrm{air}}dE}$$ (20)

if *E* is the photon energy and *E*_max_ is determined by the operating voltage of the X-ray tube.

**Figure 10:**
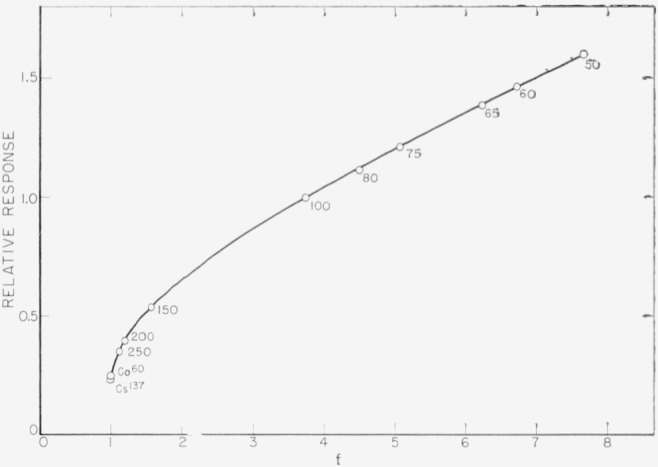
Relation between photoresponse and radiation energy absorbed per unit exposure dose rate in the photoelectric effective silicon layer. This energy is a function of ‘*f*’, the ratio of the mass energy absorption coefficients of silicon and air. Values of *f* were calculated by taking into account the spectral distribution of the radiations. Numbers near the measured points indicate the operating voltages for filtered X-rays, as referred to in table 1.

Relative values of the generated photocurrent per unit exposure dose rate are shown in figure 10 as function of *f*. The calculation of *f* was in general based on spectral distributions *J*_0_(*E*) calculated by the Kramer’s [14] method as has been done by Ehrlich and Fitch [15] for some of the investigated radiations. The calculations for the 100-kv and 150-kv X-rays were based on spectral distributions reported by Hettinger and Starfelt [16]. The influence of the absorption in the glass window on the spectral distribution was taken into account. The energy absorption coefficients of silicon were calculated from data given by White Grodstein [17] and Nelms [18]. Values of (*μ*_en_/*ρ*)_air_ were taken from the ICRU report [13]. The integration was carried out graphically by planimetry.

According to figure 10, the relative values of *I_s_* are not proportional to *f* as would be expected from eq (19). The reason for this is apparently the non-fulfillment of the electronic equilibrium condition on which eq (19) is based. The actual generation rate *g*_0_ differs from that calculated by eq (15). The ratio of the actual and calculated value of the generation rate shall be designated as *K* and be called *ionization coefficient.* Introducing *K* into eqs (15) and (19), one obtains for the actual generation rate

$$g_{0}=\frac{KE_{a}}{\epsilon v_{c}}$$ (15a)

and

$$I_{s}=2.11\times 10^{-5}R\left(v_{c}/\epsilon \right)Kf\;\mathrm{amp}.$$ (19a)

**Figure 11:**
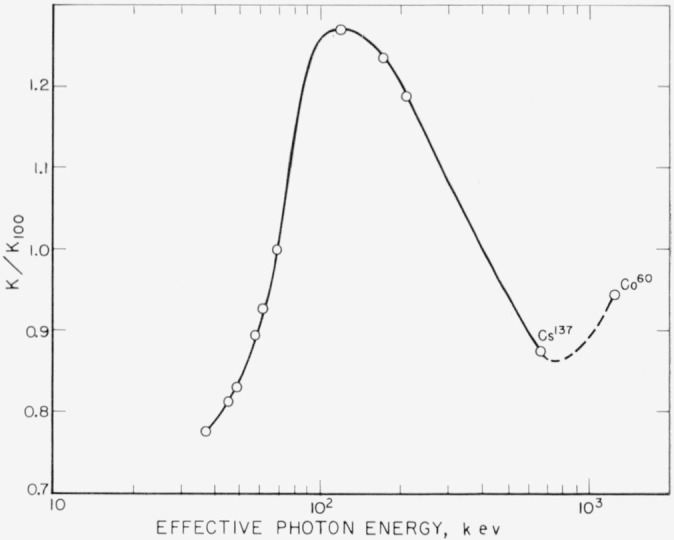
Energy dependence of relative ionization coefficient *K*/*K_100_*. *K*_100_ is the ionization coefficient for 100-kv filtered X-rays *(hv*_eff_=70 kev).

The ionization coefficient *K* is energy dependent as can be deduced from the graph in figure 10. The relative value of the generated current is, according to eq (19a),

$$\frac{I_{s}}{{\left(I_{s}\right)}_{100}}=\frac{Kf}{K_{100}f_{100}}$$ (21)

if the index 100 refers to the values for the 100-kv X-rays (*hv*_eff_=70 kev) and all values of *I_s_* are measured at the same exposure dose rate. The relative values of the ionization coefficient *K/K*_100_ can now be calculated from eq (21) by using the measured values of *I_s_*/(*I_s_*)_100_ and the calculated values of *f.* Figure 11 shows the value of *K/K*_100_ for different effective energies. It has a maximum at approximately 120 kev and decreases for smaller and higher energies. The Co^60^ point is again higher than that for Cs^137^.

It may be assumed that the ionization coefficient *K* is determined by the loss of ionization due to the deviation from the electronic equilibrium condition and by the gain in ionization from absorption inside the effective layer of Compton scattered photons produced inside and outside *v_c_.*

In the investigated energy range, the absorption of radiation takes place by photoelectric absorption and Compton scattering. In the transition range between predominant photoelectric absorption and predominant Compton scattering, the average energy of the primary electrons does not change much. This is shown in table 2 which gives for monochromatic radiations the approximate values of the fractions of energy absorbed in silicon by photoelectric absorption and Compton scattering, the average energy of photoelectrons and Compton recoil electrons, and the average energy of all electrons produced in single interactions (primary electrons). The average energy of recoil electrons was calculated as ${\bar{E}}_{c}=hv\;{\left(\sigma_{a}/\rho \right)}_{\mathrm{Si}}/{\left(\sigma_{T}/\rho \right)}_{\mathrm{Si}}$ if (*σ_T_*/*ρ*)_Si_ is the total Compton mass absorption coefficient of silicon

$${\left(\sigma_{T}/\rho \right)}_{\mathrm{Si}}={\left(\sigma_{s}/\rho \right)}_{\mathrm{Si}}+{\left(\sigma_{a}/\rho \right)}_{\mathrm{Si}}$$ (22)

and (*σ_s_*/*ρ*)_Si_ is the mass absorption coefficient referring to the energy of scattered Compton photons. The energy of photoelectrons was approximately calulated as ${\bar{E}}_{ph}=hv-I_{k}$, if *I_k_* is the ionization energy of a *K*-electron which can be assumed for silicon as 1.8 kev.

**Figure 12:**
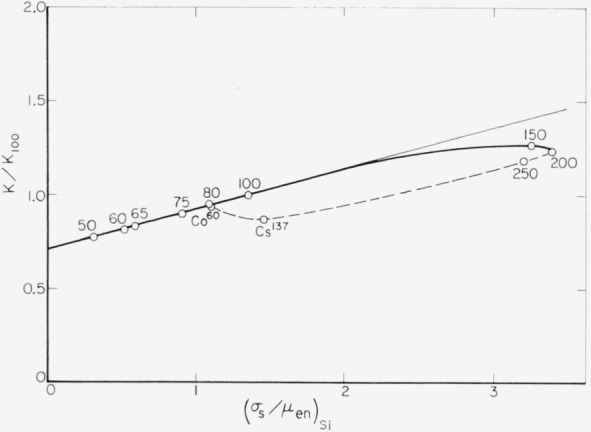
Relative ionization coefficients, *K*/*K_100_*, as a function of (*σ_s_/μ_en_*)_Si_, the ratio of energy scattered by Compton photons and energy transferred to electrons (photoelectrons and Compton recoil electrons). For the calculation of values of (*σ_s_/μ*_en_)_Si_, the spectral distributions of the radiations were taken into account. Numbers shown near the points indicate the operating voltages for X-rays (table 1).

The average electron energy changes only slightly for photon energies between 30 and 150 kev. There will thus not be much change in this range in the net loss of ionization due to high-energy electrons. It may, therefore, be assumed that the energy dependence of the ionization coefficient *K* is mainly determined by absorption of scattered Compton photons. The relative increase of the absorbed energy *E_a_* (eq (16)) due to reabsorption of scattered Compton photons will be dependent on the ratio (*σ_s_/μ*_en_)_Si_. Figure 12 shows the relative values of the ionization coefficient *K/K*_100_ as a function of (*σ_s_/μ*_en_)_Si_. The values of (*σ_s_/μ*_en_)_Si_ were calculated by taking into account the spectral distribution of the radiation as has been done in the calculation of *f* (eq (20)).

For lower energies, the values of *K/K*_100_ show a linear dependence on (*σ_s_/μ*_en_)_Si_ which has a maximum value at approximately *hv*=150 kev. For the linear part of the graph at lower energies, one can assume the relation

$$K/K_{100}=\alpha \;\left[1+\beta {\left(\sigma_{s}/\mu_{\mathrm{en}}\right)}_{\mathrm{Si}}\right]$$ (23)

with *α*=0.71 and *β*=0.29. For higher energies the *K/K*_100_ values are lower than those given by relation (23). Assuming that for very low photon energies *K*=1, one obtains by linear extrapolation to (*σ_s_/μ*_en_)_Si_=0:

$$\alpha =1/{Κ}_{100}\;\text{and}\;K=1+\beta \;{\left(\sigma_{s}/\mu_{\mathrm{en}}\right)}_{\mathrm{Si}}.$$

From the value of *α* one obtains *K*_100_=1.41. With this value, one can calculate *K* for all investigated radiations from the known values of *K/K*_100_. They increase from 1.09 for the 50-kv X-rays to a maximum value of 1.79 for the 150-kv X-rays and fall off to 1.21 for Cs^137^ gamma rays. The value of *β*=0.29 indicates that for the investigated low-energy X-rays, 29 percent of the energy of Compton photons scattered in all directions are reabsorbed in the collecting volume *v*_c_.

The collecting volume *v_c_* and the thickness *L* of the effective layer can be calculated by introducing the values of *K* in eq (19a) and calculating *I_s_* from eq (13). The energy required to produce one electron-hole pair is assumed as *ϵ*=2.25 ev, a value found by Chynoweth and McKay [19] for the electron threshold energy for electron-hole pair production in silicon. Thus, one obtains

$$v_{c}=4.4\times 10^{-2}{\mathrm{cm}}^{3}\;\text{and}\;L=5.5\times 10^{-3}\mathrm{cm}.$$

The diffusion lengths in the investigated cell were not known. However, the above value of *L*, mainly determined by the diffusion length *L_p_* of holes in the *n*-type layer, is of the same order of magnitude as usually given by other authors [20] (10^−2^ to 10^−3^ cm). Gremmelmaier [21] calculated the diffusion lengths in a silicon solar cell in a similar way from the photoresponse to Co^60^ gamma rays. However, in his calculations he used the total absorption coefficient without taking into consideration an ionization coefficient and assumed a value *ϵ* = 3.6 ev. He claimed agreement with measured values of *L_p_* within 20 percent.

## 5. Conclusions

The stable and linear response of silicon solar cells to X-rays and low-level gamma rays makes it possible to use such cells for quantitative measurements of such radiations. The maximum exposure dose rates applied in this investigation were 1.8×10^6^ r/hr for unfiltered 50 kv X-rays from a beryllium window-type tube, 6×10^4^ r/hr for unfiltered 250-kv X-rays, and 4×10^2^ r/hr for Co^60^ gamma rays. The relatively low electron energy threshold (~145 kev) for radiation damage in silicon [22] may put a limit on the use of silicon solar cells for gamma radiations of photon energies above approximately 400 kev.

The limit for a measurable response at low exposure dose rate levels is determined by the sensitivity of the measuring instruments. The response observed in this investigation for an exposure dose rate of 1 r/min was of the order of 10^−5^ volts for the open-circuit voltage and 10^−8^ amp for the photocurrent. Because of the great temperature dependence of the open-circuit voltage, it is preferable to use the photocurrent for radiation measurements. However, it will be difficult to reduce the load resistance sufficiently to make the photocurrent temperature independent because of the usual high impedance of sensitive current measuring instruments.

The response of the silicon solar cell showed a smaller directional dependence for X- and gamma rays than for visible light which follows a cosine law [7]. For Co^60^ gamma rays, the photoresponse remained constant over a wide angle of incidence of radiation.

The silicon solar cells show a better energy dependence than other solid state devices used for measurement of high-energy radiation. The ratio between the response to 50-kv X-rays (h*v*_eff_=38 kev) and that to Co^60^ gamma rays was approximately 6:1 compared with an approximate value of 25:1 for the same ratio reported for cadmium sulfide photoconductive cells [23] and photographic films [15]. Because of their high-atomic number, a high ratio may also be expected for germanium (Z_Ge_=32) and selenium (Z_Se_=34) photocells.

Silicon solar cells of the same manufacture and of the same type showed different electrical characteristics and accordingly different sensitivity with the same measuring setup. The measurements reported in this investigation were all carried out on one and the same cell.

The response of silicon solar cells to X- and gamma rays could be improved by making certain adjustments to the cell, which is actually designed for use with visible light. In high energy radiation cells, the *p*-type and *n*-type layers should be larger than the diffusion lengths by an amount which is equal to the range of the most energetic electron produced by the radiation. In this way electronic equilibrium condition could be approached. The ionization coefficient and the collecting volume would thus be increased and the cell would become more sensitive for high-energy radiation. A special design of the cell casing could improve the directional and energy dependence of the photoresponse. The overall sensitivity could be increased by using multicell arrangements and stacking of cells in case of irradiation with high-energy gamma rays. The silicon solar cell could be specially useful in monitoring and relative measurements of radiation, having also the advantage of being independent of an external electric power supply.

## Figures and Tables

**Table 1:** Qualities of investigated radiations

Constant X-ray tube voltage	Filtration^a^ (mm)			Approximate half-value layer (HVL)	Approximate effective photon energy (h*v*_eff_)

X-rays					

*kv*	Cu	Sn	Pb	*mm* Cu	*kev*
50	………	………	0.125	0.16	38
60	0.32	………	.125	.26	46
65	.32	………	.125	.31	49
75	.81	………	.125	.47	58
80	………	………	.52	.55	62
100	………	………	.52	.72	70
150	4.00	1.53	………	2.4	122
200	.59	4.00	.69	4.1	170
250	.62	1.00	2.67	5.4	210

Gamma rays					

Cs^137^					661
Co^60^					1250

a Additional to inherent tube filtration of 3 mm Al.

**Table 2:** Average energies of primary electrons produced by radiation in silicon in single interactions

Photon energy h*v*	Fraction of energy absorbed		Average electron energy		
	
	Photoelectrons	Compton-electrons	Photoelectrons	Compton-electrons	Total of photo- and Compton-electrons
					
*kev*	%	%	*kev*	*kev*	*kev*
30	99.3	0.7	28.2	1.5	24.6
40	97.6	2.4	38.2	2.6	28.4
50	94.3	5.7	48.2	4.0	29.1
100	53.8	46.2	98.2	13.8	25.5
150	20.5	79.5	148.2	27.2	32.6
200	8.8	91.2	198.2	43.6	46.8
250	5.5	94.5	248.2	62.0	64.6

## Footnotes

2 Figures in brackets indicate the literature references at the end of this paper.

3 Type S–1A cells manufactured by Hoffman Electronics Corp. (Semiconductor Division).

4 The calibrations of the *r*-meters were carried out by Mr. LeRoy DeLa Vergne of the Radiological Equipment Section of the National Bureau of Standards.

5 The influence of acceptor and donor impurities on the absorption coefficient was assumed as negligible because of their very small concentration.
